# Hemodynamic impact of acute liver injury on cardiac function: An in silico study via a closed-loop cardiovascular model

**DOI:** 10.1371/journal.pcbi.1014006

**Published:** 2026-02-24

**Authors:** Jiyang Zhang, Zhongyou Li, Lin Feng, Jialu Zhang, Taoping Bai, Wentao Jiang

**Affiliations:** 1 Department of Mechanical Science and Engineering, Sichuan University, Chengdu, China; 2 Sichuan Province Biomechanical Engineering Laboratory, Chengdu, China; 3 Department of Biomedical Engineering, Faculty of Engineering, The Hong Kong Polytechnic University, Hong Kong, China; 4 College of Computer Science, Sichuan Normal University, Chengdu, China; University of Notre Dame, UNITED STATES OF AMERICA

## Abstract

Acute liver injury and cardiovascular disease interact, forming a mutually exacerbating vicious cycle. However, the dynamic influence of hepatic vascular impedance on cardiac function has not been systematically elucidated. To address this gap, a closed-loop hemodynamic model based on lumped parameters was developed, encompassing the heart, liver, and the systemic arterial and venous circulation. This model was used to analyze how alterations in hepatic vascular impedance influence cardiac function and to provide a theoretical foundation for understanding liver–heart comorbidities. Healthy subjects served as the control group, while acute liver injury was simulated by proportionally increasing hepatic microvascular resistance. Changes in cardiovascular hemodynamic parameters were then systematically compared across conditions. As the severity of acute liver injury increases, the peak aortic flow and total cardiac output significantly decrease, with stroke volume reduced by approximately 17%. The left ventricular end-diastolic volume and stroke work are markedly diminished. Effective arterial elastance increases by about 20.7%, and the left ventricular ejection fraction decreases by approximately 4%. Furthermore, the change in hepatic arterial flow is considerably greater than that in portal vein flow. This closed-loop hemodynamic model reveals that acute liver injury leads to a reduction in preload and an increase in afterload, thereby causing abnormalities in both systolic and diastolic cardiac function. Global sensitivity analysis demonstrated that changes in presinusoidal vascular resistance serve as the major contributors to the resulting cardiac dysfunction. These findings provide a theoretical basis for understanding the interplay between liver and heart, and offer a feasible method for pre-assessing cardiovascular risk in patients prior to liver resection or transplantation.

## Author summary

The liver and heart are intimately connected through blood circulation, yet how liver damage directly impacts cardiac function has remained unclear. The liver has a unique dual blood supply (from both the hepatic artery and portal vein), and when injured, its blood vessels stiffen and narrow, forcing the heart to work harder. In this study, we used a computational model of the human circulatory system to investigate how acute liver injury—such as from infections, toxins, or surgery—affects the cardiac function. Our simulations revealed that as acute liver injury worsens, blood flow to the heart decreases, while resistance against pumping increases. This double burden weakens the heart, reducing its pumping efficiency by nearly 20% in severe cases. These findings help explain why liver disease patients often develop heart complications and why heart risks must be carefully assessed before liver surgeries or transplants. By uncovering this liver-heart interaction, our research provides a foundation for better treatments and highlights the need for integrated care in patients with both liver and heart conditions.

## 1. Introduction

The liver is the largest solid organ in the human body, playing a critical role in metabolism, detoxification, protein synthesis, and nutrient storage. Hepatic injury encompasses a diverse range of pathological states, including acute liver injury, chronic hepatitis, and cirrhosis, and can lead to irreversible tissue damage, such as damage to sinusoidal endothelial cells, microthrombosis, and even fibrosis [1,2]. Recent studies have demonstrated a complex bidirectional pathological relationship between liver injury and cardiovascular diseases (CVD). For instance, alcoholic liver cirrhosis increases the risk of left ventricular diastolic dysfunction [3], and patients often exhibit compensatory cardiac dysfunction, which in turn further impairs the liver’s metabolic and detoxification capacities, thereby exacerbating disease progression [4]. In patients with acutely decompensated heart failure, the level of the fibrosis marker P4NP 7S in the liver is significantly correlated with right atrial pressure [5]. Moreover, patients with nonalcoholic fatty liver disease have a 64% higher risk of experiencing fatal or non-fatal CVD [6]. Although clinical correlations between liver and cardiac dysfunction are well-established, the mechanistic understanding of their interplay remains limited [7]. Current research predominantly relies on clinical observations and animal models, yet the underlying hemodynamic mechanisms driving this relationship remain poorly characterized [7–10].

Hemodynamic studies have shown that changes in vascular impedance are closely related to the occurrence of CVD. An increase in vascular impedance can lead to an early reflection of the pulse wave, thereby elevating aortic systolic pressure and increasing the risk of CVD [11–14]. Age-related increases in vascular impedance can impair diastolic function, thereby raising the incidence of diastolic heart failure [15,16]. Increased pulmonary impedance leads to abnormal myocardial systolic and diastolic functions and a reduction in cardiac reserve, ultimately resulting in diminished cardiac performance [17]. The decrease in the impedance of the epidermal blood vessels caused by heat exposure will reduce the visceral perfusion [18,19].

Building on the established link between vascular impedance and CVD, acute liver injury-induced increases in hepatic impedance cause cardiac dysfunction. Specifically, acute liver injury damages hepatic sinusoidal capillaries and triggers coagulation abnormalities, vascular shunting, and tissue fibrosis, thereby raising hepatic impedance [20–22]. These pathological alterations impose an increased hemodynamic burden on the heart, particularly in patients undergoing liver transplantation or resection [23,24]. Thus, understanding the hemodynamic impact of acute liver injury on cardiac function is crucial, as it not only aids in the development of precise therapeutic strategies but also assists clinicians in performing comprehensive preoperative cardiovascular assessments, predicting postoperative complications, and implementing measures to mitigate surgical risks [23].

Numerous computational modeling studies have investigated hepatic hemodynamics. Mazumder et al. [25] builds a computational model of portal hypertension which supports that patient-level differences in Portal Venous Remodeling may explain disparate clinical trajectories of disease. Rojas et al. [26] simulated the hemodynamic consequences of vascular fibrosis within the liver using a one-dimensional blood flow model. Audebert et al. [27] based on porcine hepatectomy experimental data, proposed a numerical scheme for a one-dimensional hemodynamic model to investigate the physiological changes observed during liver resection. Golse et al. [28] further introduced a digital twin framework to predict post-hepatectomy portal hypertension, providing a clinical proof of concept for patient-specific hemodynamic prediction. Wang et al. [29,30] employed a stochastic lumped-parameter model to perform global sensitivity analysis of hepatic venous pressure gradient (HVPG) measurements, identifying presinusoidal portal vascular resistance and splanchnic vascular resistance were the major factors determining the relative difference between HVPG and portal pressure gradient.

Based on this, we establish a full circulation model incorporating the dual blood supply system of the liver to systematically analyze the impact of hepatic vascular impedance changes on cardiac function. This model aims to deepen the understanding of the pathogenesis of CVD following acute liver injury and to provide data support for clinical diagnosis, treatment, and surgical decision-making.

## 2. Methods

**Fig 1:**
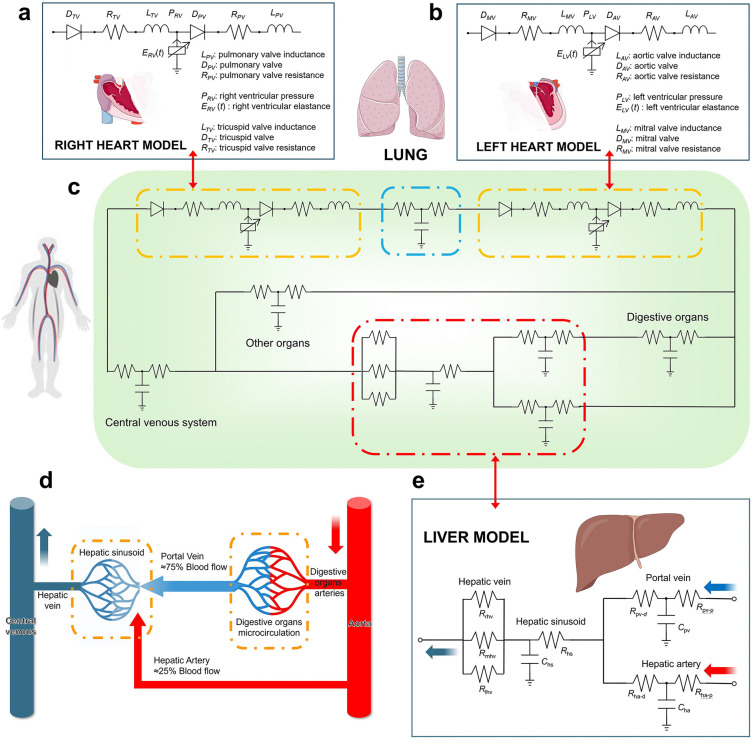
Closed-loop cardiovascular LPM: **(a)** Right heart model; **(b)** Left heart model; **(c)** Closed-loop systemic circulation model; **(d)** Schematic of the dual hepatic blood supply (red indicates oxygen-rich blood, and blue represents blood with low oxygen content.); **(e)** Lumped-parameter representation of the hepatic circulation. Some icons were adapted from Bioicons (MIT License, https://bioicons.com/).

We have developed a closed-loop cardiovascular lumped parameter model (LPM) with a series of electrical components that incorporates the dual blood supply system of the liver and can control hepatic impedance. This framework enables simulation of systemic circulatory effects induced by varying degrees of acute liver injury. The model integrates the heart, liver, lung, digestive organs, other organs, and the venous system (Fig 1). The functions of each circuit element and their governing equations are described in the following sections and in S1 Text.

### 2.1 Heart model

The cardiac model in this study divides the heart into left and right compartments with identical structural configurations. Taking the left heart as an example, the model includes key hemodynamic components: Diodes (*D*_AV_: aortic valve; *D*_MV_: mitral valve, functioning to prevent blood reflux, with pathological regurgitation conditions such as valvular calcification excluded, and model the pressure drop across a valve under unidirectional flow using a nonlinear hyperbolic-tangent resistor [31]); Resistances (*R*_AV_: aortic valve resistance; *R*_MV_: mitral valve resistance, representing hemodynamic resistance caused by valve morphology and fluid dynamics); and Inductances (*L*_AV_: aortic valve inductance; *L*_MV_: mitral valve inductance, which characterize the inertial effects of blood flow after valve passage, reflecting the dynamic response during acceleration and deceleration). The cardiac structure and parameters were based on the work of Arthurs et al. [32]. These parameters were kept constant during the subsequent optimization process (Table 1).

**Table 1:** Parameters of left and right heart components.

Left heart	*R*_MV_	*L*_MV_	*R*_AV_	*L*_AV_
	Pa*·* s*·* mm ^*−* 3^	Pa· s^2^· mm ^− 3^	Pa*·* s*·* mm ^*−* 3^	Pa· s^2^· mm ^− 3^
	3.9 *×* 10 ^*−* 4^	1 *×* 10 ^*−* 5^	1 *×* 10 ^*−* 5^	1 *×* 10 ^*−* 5^
**Right heart**	***R***_**TV**_	***L***_**TV**_	***R***_**PV**_	***L***_**PV**_
	Pa*·* s*·* mm ^*−* 3^	Pa· s^2^· mm ^− 3^	Pa*·* s*·* mm ^*−* 3^	Pa· s^2^· mm ^− 3^
	1 *×* 10 ^*−* 5^	1 *×* 10 ^*−* 5^	1 *×* 10 ^*−* 5^	1 *×* 10 ^*−* 5^

Ventricular pressure and volume changes during systole and diastole can be represented using the time-varying elastance model proposed by Suga et al. [33], as shown in Equation (1):


$$P(t)=E(t)\cdot [V(t)-V_{\text{0}}]$$ (1)

**Fig 2:**
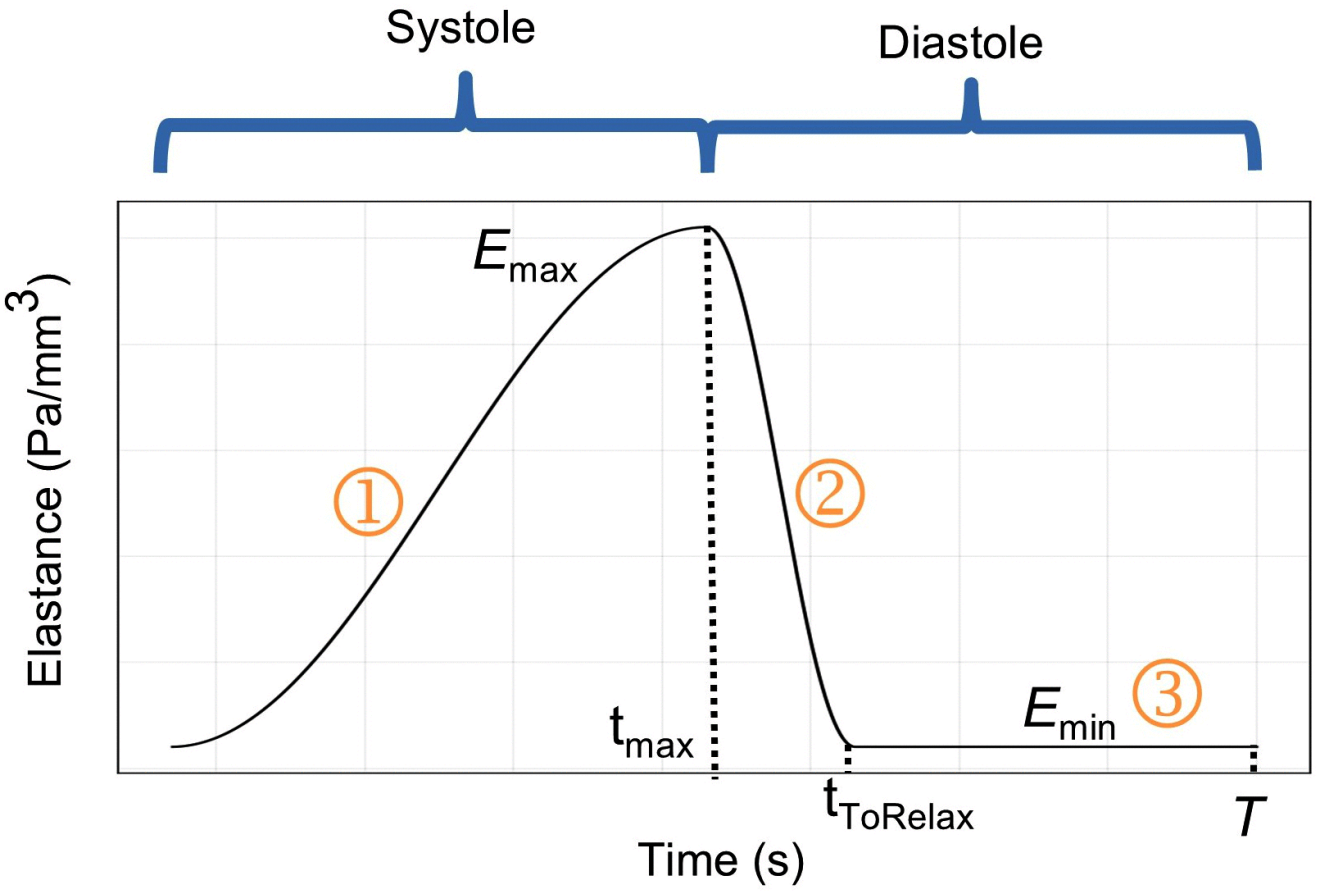
Triphasic piecewise elastance function: myocardial contraction; myocardial relaxation and complete myocardial relaxation.


In this model, time-varying elastance *E*(*t*), is treated as a time-dependent function. *E*(*t*) represents the ratio of ventricular pressure *P*(*t*) to volume changes [*V*(*t*)-*V*_0_], capturing the dynamic stiffness of the ventricle during both systole and diastole (Fig 2). *V*_0_ is the unstressed ventricular volume, corresponding to the volume at which ventricular pressure is zero. In this study, *V*_0_ of both the left and right ventricles were set to 10 mL [34]. Within a certain range, *E*(*t*) exhibits load-independence, meaning it reflects the intrinsic contractile capability of the ventricle. In this study, *E*(*t*) was kept fixed to represent unaltered myocardial contractility under acute conditions. *E*(*t*) is governed by a triphasic piecewise function as follows [34]:

$$E\left(t\right)=\left\{ \begin{array}{cc} @ll@E_{min}+0.5\times (E_{max}-E_{min})\times \left(1-cos\left(\frac{\pi t}{t_{max}}\right)\right), & 0<t\leq t_{max}\\ E_{min}+0.5\times (E_{max}-E_{min})\times \left(1+cos\left(\frac{\pi (t-t_{max})}{t_{\text{ToRelax}}}\right)\right), & t_{max}<t\leq t_{max}+t_{\text{ToRelax}}\\ E_{min}, & t_{max}+t_{\text{ToRelax}}<t\leq T \end{array}\right.$$ (2)

where *E*_max_: maximum elastance; *E*_min_: minimum elastance; *T*: cardiac cycle; *t*_max_: time to maximum elastance; *t*_ToRelax_: time from maximum to minimum elastance. The left and right ventricles have different elastance values (*E*_max_ and *E*_min_) during optimization, while the three timing parameters (*T*, *t*_max_, *t*_ToRelax_) are the same for both ventricles (Table 2).

**Table 2:** Parameters of the elastance function for the left and right ventricles.

*E*_maxL_	*E*_minL_	*E*_maxR_	*E*_minR_	*T*	*t*_max_	*t*_ToRelax_
Pa*·* mm ^*−* 3^	Pa*·* mm ^*−* 3^	Pa*·* mm ^*−* 3^	Pa*·* mm ^*−* 3^	s	s	s
0.394	9e-5	0.152	9e-5	0.684	0.377	0.104

### 2.2 Liver model

As shown in Fig 1d, the liver has a unique dual blood supply system, provided by the hepatic artery and the portal vein. The hepatic artery, characterized by higher blood pressure, delivers approximately 25% of the hepatic blood supply, which is rich in oxygen. In contrast, the portal vein, with lower blood pressure, supplies about 75% of the blood, which is rich in nutrients and metabolic waste but has lower oxygen content [35]. The blood from the hepatic artery and the portal vein converges in the hepatic sinusoids. After being processed by the liver, the blood exits through the hepatic veins and eventually returns to the heart, completing the circulatory cycle. In Fig 1e, the liver is represented using the following components: *R*_pv-p_ and *R*_pv-d_, representing the proximal and distal portal vein resistances, respectively; *C*_pv_, the portal vein compliance; *R*_ha-p_ and *R*_ha-d_, the proximal and distal hepatic artery resistances, respectively; *C*_ha_, the hepatic artery compliance; *R*_hs_ and *C*_hs_, the hepatic sinusoidal resistance and compliance, respectively; and *R*_lhv_, *R*_mhv_, and *R*_rhv_, representing the resistances of the left, middle, and right hepatic veins.

### 2.3 Closed-loop cardiovascular model

The lungs, systemic other organs, and venous system are modeled using a three-element RCR LPM [36,37], forming a closed-loop circulatory system. The parameter values are listed in Table 3 [27], and the governing equations are provided in S1 Text.

**Table 3:** Parameters of closed-loop LPM.

Vessels	*R*_*p*_	*C*	*R*_*d*_
	Pa·s·mm ^− 3^	mm^3^·Pa ^− 1^	Pa·s·mm ^− 3^
Portal vein	0.00318	1.200	0.03184
Hepatic artery	0.17755	0.630	1.84015
Hepatic sinusoid	–	1.000	0.00884
Left hepatic vein	0.00001	–	–
Right hepatic vein	0.00001	–	–
Middle hepatic vein	0.00001	–	–
Lung	0.00027	1.000	0.00265
Digestive organs	0.05607	0.630	0.58110
Other organs	0.01869	6.302	0.19370
Central venous system	0.00014	12.000	0.00142

The model (Fig 1c) is implemented in CRIMSON, an open-source software platform for patient-specific hemodynamic simulations [38].

### 2.4 Simulation setup

The numerical simulations were performed using the solver embedded in CRIMSON under closed-loop purely 0D configuration. A time step size of 0.001 s and a total of 8,000 time steps were implemented. After confirming periodic stability (defined as less than 1% variation in cycle-averaged hemodynamic parameters between consecutive cycles), data from the final two cardiac cycles were extracted as the simulation results. CRIMSON employed a second-order accurate generalized-α method for time discretization, with the residual set to 0.001 for numerical integration.

### 2.5 Parameter calibration and optimization

The final LPM parameters were calibrated based on clinically informed reference values, ensuring agreement with normative hemodynamic parameters listed in Table 4 for healthy individuals [39,40].

**Table 4:** Physiological aortic pressure-flow parameters in healthy adults.

$Q_{Aorta}^{Mean}$	$Q_{Aorta}^{Max}$	$P_{Aorta}^{Min}$	$P_{Aorta}^{Max}$
(L/min)	(L/min)	(mmHg)	(mmHg)
5 [40]	30 [39]	80 [39]	120 [40]

Based on previously reported estimation methods and pig-specific physiological reference values reported by Audebert et al. [27], an initial parameter set was selected for optimization. For the RCR Windkessel model, the distal-to-proximal resistance ratio was initialized at 10:1 [27], and all parameters were treated as free variables during optimization. The cardiac subsystem consisted of seven parameters (*E*_maxL_, *E*_minL_, *E*_maxR_, *E*_minR_, *T*, *t*_max_, *t*_ToRelax_), while three scaling coefficients governed the peripheral circulation: the distal resistance scaling coefficient (*A*_1_), proximal resistance scaling coefficient (*A*_2_), and vascular compliance scaling coefficient (*A*_3_). Each parameter was constrained within its physiologically plausible range, shown in Table 5.

**Table 5:** Parameter search ranges.

Parameter	*A*_1_	*A*_2_	*A*_3_	*E*_maxL_	*E*_minL_	*E*_maxR_	*E*_minR_	*T*	*t*_max_	*t*_ToRelax_
Search Range	0.1 – 10	0.1 – 10	0.1 – 10	1e-5 – 1	1e-5 – 1	1e-5 – 1	1e-5 – 1	0.5 – 2	0.1 – 0.5	0.05 – 0.2

The optimization was performed using the Nelder–Mead simplex algorithm implemented in SciPy optimization library (scipy.optimize.minimize) [41]. The loss function was defined as:


$$L=\left|\frac{Q_{Aorta}^{Mean}-5}{\text{5}}\right|+\left|\frac{P_{Aorta}^{Max}-120}{120}\right|+\left|\frac{P_{Aorta}^{Min}-80}{80}\right|+\left|\frac{Q_{Aorta}^{Max}-30}{30}\right|$$ (3)

where, $Q_{Aorta}^{Mean}$: Mean aortic flow; $P_{Aorta}^{Max}$: Aortic systolic pressure; $P_{Aorta}^{Min}$: Aortic diastolic pressure; $Q_{Aorta}^{Max}$: Peak aortic flow. Including these terms ensures that the model reproduces physiologically realistic pressures and flow profiles. The maximum number of function evaluations was set to 300. Convergence tolerances were defined as follows: the tolerance for changes in the objective function and the tolerance for changes in the parameter vector were both set to 1 × 10 ⁻ ⁴. The optimization terminated when either of these criteria was satisfied, i.e., once successive iterations produced changes in both the objective function and the parameter values below the specified thresholds.

**Fig 3:**
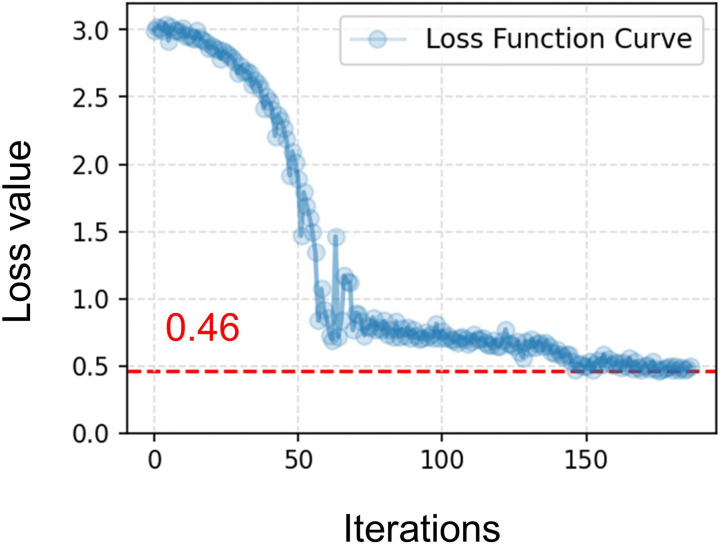
Loss function value curve.

To mitigate the risk of convergence to local minima, a multi-initialization strategy was adopted. Several distinct initial guesses were tested, and the resulting optimized parameter sets were compared to verify that none of the variables approached their predefined boundary limits. Among all optimization runs, the solution with the lowest loss was selected as the final model output. The optimization converged to a minimum loss of 0.46 (Fig 3), and the corresponding parameter set was designated as the healthy baseline configuration. The simulated cardiac cycle duration was determined to be 0.684 s (87.7 bpm), a value that falls within the normal physiological range of 60–90 bpm reported by David et al [42]. This fixed heart rate was subsequently applied to all following analyses to ensure consistency across simulation conditions. After calibrating the cardiac and systemic peripheral impedance parameters, the resistance ratio between the portal vein and hepatic artery was manually adjusted to satisfy the portal–hepatic venous flow distribution described above.

### 2.6 Liver injury grouping

Liver injury leads to a series of pathophysiological effects, including sinusoidal endothelial cell damage, coagulation activation, microvascular obstruction, tissue fibrosis, vascular stiffening, sinusoidal compression, and increased intrahepatic vascular resistance [22,43,44]. Based on the assumption that these effects uniformly impact the hepatic vascular bed, we simulated varying degrees of liver injury by proportionally scaling microvascular resistance parameters. Baseline resistance values for healthy controls (M1) were calibrated as *R*_pv-d_ = 0.03184 Pa*·*s*·*mm^*−*3^, *R*_ha-d_ = 1.84015 Pa*·*s*·*mm^*−*3^, and *R*_hs_ = 0.00884 Pa*·*s*·*mm^*−*3^. Based on the typical effects of liver injury on portal pressure, which is controlled to be below 20 mmHg [45], this study defines the experimental groups (M5, M10, M15, M20) by multiplying these resistance parameters by factors of 5, 10, 15, and 20, respectively, to represent varying degrees of acute liver injury.

### 2.7 Sensitivity analysis method

Variance-based global sensitivity analysis [46] was employed to quantitatively evaluate the influence of hepatic vascular properties on cardiac hemodynamics, including cardiac output (CO), mean aortic pressure (MAP), and stroke work (SW). Four hepatic parameters were selected for this analysis: *R*_pv-d_, *R*_ha-d_, *R*_hs_, and hepatic venous resistance *R*_hv_. Each parameter was scaled relative to its baseline value according to:


$$x_{ij}=x_{j,baseline}\cdot scale_{ij},\;j\in \{R_{pv\_d},R_{ha\_d},R_{hs},R_{hv}\},\;i=1,2,\ldots ,n$$ (4)

where $x_{ij}$ represents the *j*-th parameter in the *i*-th sample, $x_{j,baseline}$ is the baseline value, and ${scale}_{ij}$ is the sampling factor generated using Saltelli’s quasi-random sequence [47]. The parameter bounds for the sensitivity analysis were defined relative to their baseline values. Specifically, the scaling factors for all four hepatic parameters were restricted to the interval [0.5, 10], allowing the parameters to vary from half to ten times their baseline values, thereby capturing both mild and severe deviations from normal physiology. For each parameter set, the cardiovascular model was simulated to compute CO, MAP, and SW.

Two sensitivity indices can be obtained from the analysis: the first-order sensitivity index ($S_{j}$) and the total-order sensitivity index ($ST_{j}$). The first-order index measures the averaged effect of variations in a single input parameter on the variance of the model output, while the total-order index quantifies the integrative contribution of the parameter and its interactions with all other parameters [48]. Theoretically, a first-order index approaching its corresponding total-order index indicates a strong independent effect of the parameter on model output, whereas a large difference between the two indicates that the parameter’s influence is highly dependent on interactions with other parameters. In this study, $S_{j}$ and $ST_{j}$ were calculated for each hepatic parameter using Saltelli’s approach [47]. Mathematically, the indices were computed as:


$$S_{j}=\frac{V(E(y|x_{j}))}{V(y)},\;ST_{j}=1-\frac{V(E(y|x_{-j}))}{V(y)}$$ (5)

where V(y) is the unconditional variance of the output y, $E\left(y|x_{j}\right)$ denotes the expectation of y conditional on the *j*-th parameter, and $x_{-j}$ includes all parameters except the *j*-th. A total of N×(2K+2)=500×(2×4+2)=5000 model evaluations were performed, allowing a quantitative ranking of the hepatic parameters according to their independent and interaction effects on systemic hemodynamics under variable hepatic vascular conditions.

## 3. Results

The following sections present the simulation results of the closed-loop cardiovascular model with increasing severity of acute liver injury. Hemodynamic changes are examined in terms of systemic blood flow and pressure, portal venous contributions, left ventricular pressure–volume relationships, and the sensitivity of cardiac function to key hepatic vascular parameters.

### 3.1 Blood flow

**Fig 4:**
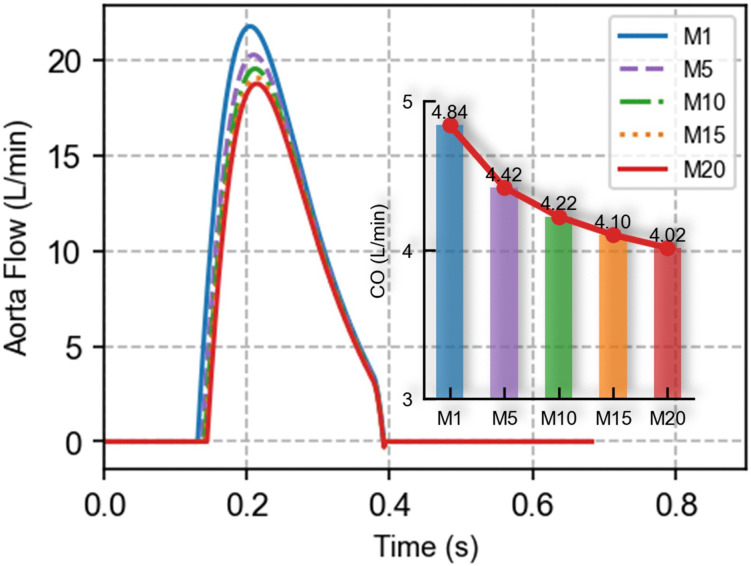
Impact of acute liver injury on aortic flow waveform and cardiac output.

As shown in Fig 4, the progressive increase in hepatic vascular resistance led to a decline in peak aortic flow and a delayed onset of systolic ejection within the cardiac cycle. This resulted in a reduction of CO from 4.84 L/min (M1) to 4.02 L/min (M20), representing a 16.94% decrease.

**Fig 5:**
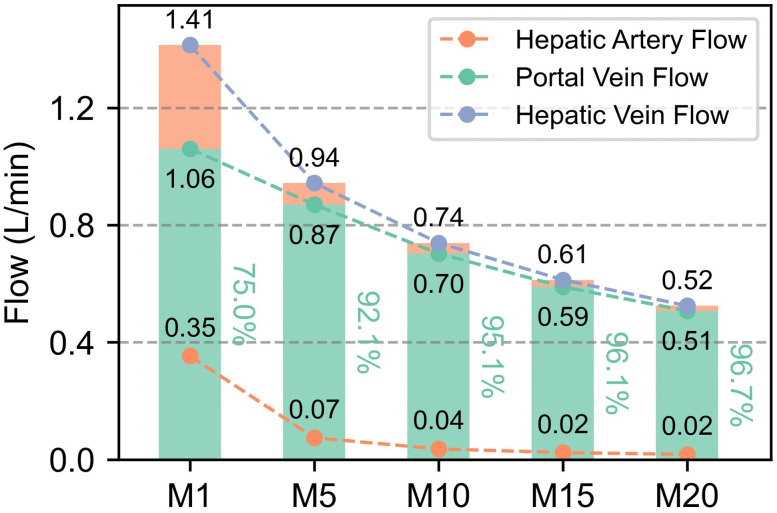
Hepatic vein flow contribution.

As shown in Fig 5, total hepatic vein flow (portal vein flow + hepatic arterial flow) decreased markedly with increasing acute liver injury, declining from 1.41 L/min in M1 to 0.52 L/min in M20 (≈63.1% reduction). Specifically, portal vein flow dropped from 1.06 L/min in M1 to 0.51 L/min in M20 (≈51.9% reduction), while hepatic arterial flow plummeted from 0.35 L/min in M1 to 0.02 L/min in M20 (≈94.3% reduction). Notably, despite concurrent reductions in absolute flow of both vessels, hepatic vascular resistance exerted a stronger inhibitory effect on arterial flow, evidenced by the dramatic decline in arterial contribution to hepatic venous flow from 25.0% in M1 to 3.3% in M20, whereas portal vein contribution increased from 75.0% to 96.7%.

### 3.2 Blood pressure

**Fig 6:**
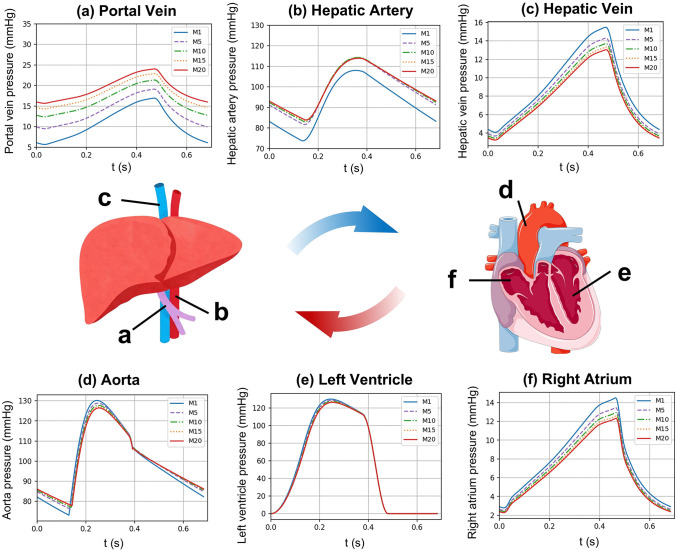
Pressure waveform variations in key cardiac and hepatic regions: (a) portal vein; (b) hepatic artery; (c) hepatic vein; (d) aorta; (e) left ventricle and (f) right atrium. Some icons were adapted from Bioicons (MIT License, https://bioicons.com/).

**Fig 7:**
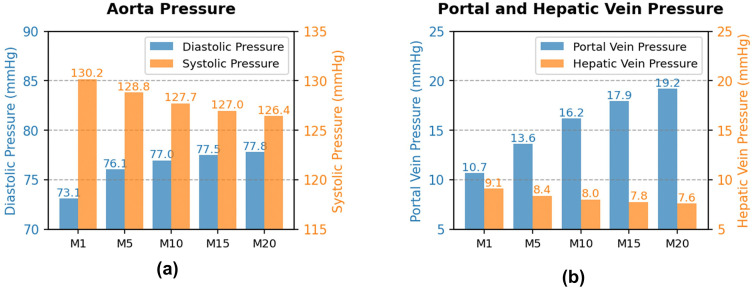
Pressure changes under acute liver injury: (a) Systolic and diastolic blood pressures; (b) Portal and hepatic vein pressures.

Figs 6 and 7 illustrate the hemodynamic waveform and numerical changes in key hepatic and cardiac regions during progressive acute liver injury. In hepatic hemodynamics, the portal vein exhibited a flattened pulsatile waveform, with its peak pressure increasing progressively as liver injury worsened. The mean portal vein pressure rose significantly from 10.7 mmHg (M1) to 19.2 mmHg (M20), which increased by 79.4%. Concurrently, the mean hepatic vein pressure decreased from 9.1 mmHg (M1) to 7.6 mmHg (M20), aligning with the downward trend in peak pressure. The hepatic arterial pressure waveform displayed characteristic arterial pulsatility, featuring distinct systolic and diastolic peaks and troughs. Hepatic artery pressure demonstrated high sensitivity to liver injury: significant waveform alterations in amplitude and morphology occurred during the transition from M1 to M5. Further progression of acute liver injury (M5 to M20) showed in stabilized waveform changes, with reduced intergroup variability, though remaining markedly distinct from controls.

In cardiac hemodynamics, aortic systolic blood pressure (SBP) progressively declined (130.2 mmHg at M1 → 126.4 mmHg at M20), while diastolic blood pressure (DBP) steadily increased (73.2 mmHg at M1 → 77.8 mmHg at M20). The right atrial peak systolic pressure decreased, and the waveform exhibited an overall downward trend.

### 3.3 P-V loop

**Fig 8:**
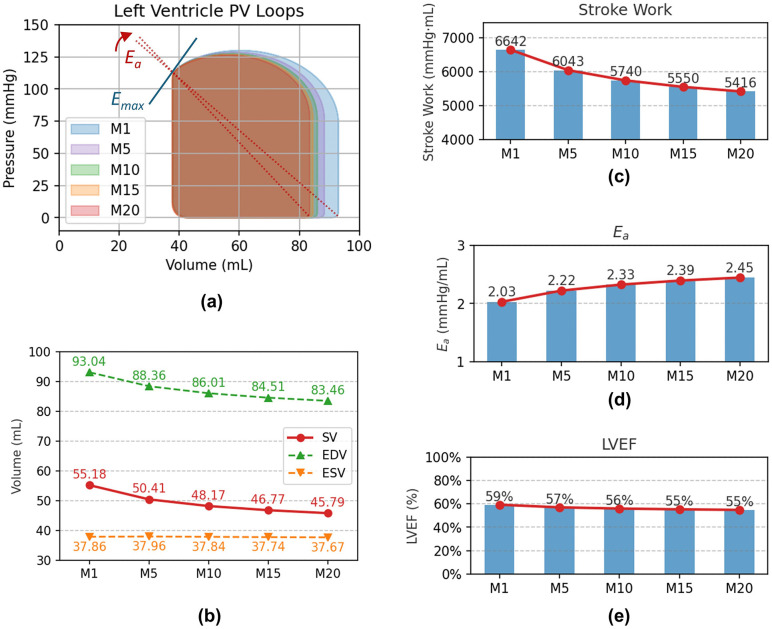
Cardiac function of the left heart ventricle: (a) PV-loop (b) EDV, ESV and SV (c) SW (d) LVEF.


The pumping function of the heart primarily relies on ventricular filling during diastole and ejection during systole. Fig 8 illustrates the dynamic relationship between pressure and volume in the left ventricle within one cardiac cycle, depicted by the pressure-volume (PV) loop. With increasing severity of acute liver injury, the end-diastolic volume (EDV) gradually decreases, while the end-systolic volume (ESV) remains relatively stable. This change indicates a significant reduction in stroke volume (SV), which is the volume of blood ejected by the heart during each contraction. SV is calculated as:

SV=EDV−ESV (6)

From M1 to M20, SV decreased from 55.18 mL to 45.79 mL (a 17.0% reduction).

Stroke work represents the work performed by the heart during each contraction and is measured by the area enclosed within the PV-loop [49,50]. With increasing severity of acute liver injury, SW decreased from 6642 mmHg·mL at M1 to 5416 mmHg·mL at M20 (a 18.5% reduction). This reduction not only reflects the decrease in SV but is also associated with increased afterload. The increase in afterload is driven by elevated effective arterial elastance (*E*_*a*_), which is calculated as:


$$E_{a}=\frac{ESP}{SV}$$ (7)

where ESP is the end-systolic pressure (about 112 mmHg in this study). From M1 to M20, *E*_*a*_ increased from 2.03 mmHg/mL to 2.45 mmHg/mL (a 20.7% increase). End-systolic elastance (*E*_*es*_) was defined as the slope of the end-systolic pressure–volume relationship (ESPVR). In this study, *E*_*es*_ corresponds to *E*_*max*_ and is treated as a fixed value; therefore, it does not change in the Fig 8a with increasing severity of acute liver injury.

Left ventricular ejection fraction (LVEF) is a key indicator of cardiac pumping efficiency, representing the proportion of blood ejected from the ventricle relative to its total volume. It is calculated as:


$$LVEF=\frac{EDV-ESV}{EDV}\times 100\%$$ (8)

From M1 to M20, LVEF decreased from 59% to 55%.

### 3.4 Sensitivity indices of model parameters

**Fig 9:**
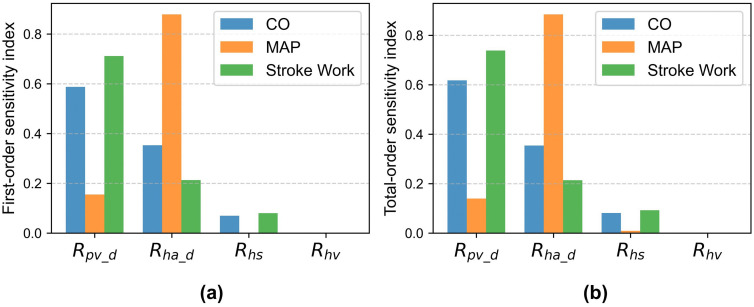
Results of the variance-based global sensitivity analysis: (a) first-order sensitivity index, (b) total-order sensitivity index. The sensitivity indices are evaluated for four hepatic vascular parameters with respect to CO, MAP and SW, respectively. Portal vein distal resistance (*R*_*pv_d*_), hepatic artery distal resistance (*R*_*ha_d*_), sinusoidal resistance (*R*_*hs*_), and hepatic venous resistance (*R*_hv_).

Based on the processed Sobol analysis results, the sensitivity indices of the hepatic vascular parameters on systemic hemodynamics are summarized in Fig 9. The results indicate that CO was most sensitive to *R*_pv_d_ (*S* = 0.59, *ST* = 0.62), followed by *R*_ha_d_, whereas *R*_hs_ and *R*_hv_ had minimal influence. SW was predominantly influenced by *R*_pv_d_ (*S* = 0.71, *ST* = 0.74), with secondary contributions from *R*_ha_d_, while *R*_hs_ and *R*_hv_ again showed negligible effects. In contrast, MAP was highly sensitive to *R*_ha_d_ (*S* = 0.88, *ST* = 0.88), whereas *R*_pv_d_ and *R*_hs_ had moderate impact and *R*_hv_ remained insignificant. Overall, the close agreement between first-order and total-order indices for each parameter suggests that the independent effects of these hepatic parameters dominated, with only minor interaction effects.

## 4. Discussion

This study developed a closed-loop cardiovascular LPM employing electrical analog components to simulate the systemic circulation and control hepatic impedance. Increased hepatic microvascular resistance delayed systolic ejection and reduced CO by 16.9%. Meanwhile, portal vein pressure nearly doubled, while left ventricular SV, LVEF, and SW decreased, and *E*_a_ increased. These simulation results demonstrate that acute liver injury markedly impairs cardiac function through complex hemodynamic alterations, and the impairment worsens with increasing severity of acute liver injury.

Numerical simulations revealed that increased microvascular resistance due to acute liver injury not only directly altered intrahepatic blood flow distribution but also indirectly impaired cardiac systolic and diastolic functions through reduced venous return and altered hemodynamic loading. Diminished SBP results from decreased CO, whereas elevated DBP arises from augmented residual blood volume post-aortic valve closure. These findings validate the “liver-heart axis” theory proposed in cirrhotic cardiomyopathy studies [51,52], and align with clinical observations of reduced CO and hemodynamic abnormalities in hepatic dysfunction [53]. With increasing severity of acute liver injury, portal hypertension and splanchnic congestion develop [54]. This reduces venous return, limits ventricular filling, and subsequently decreases right atrium pressure. Reduced preload and increased afterload lead to declines in SV, SW, and LVEF. Although the LVEF reduction is modest (4%), this trend suggests a mild impairment in cardiac pumping function. The increase in *E*_a_ indicates a significant rise in arterial resistance to cardiac ejection. Notably, while our simulations demonstrated decreased CO, clinical studies often report hyperdynamic circulation in cirrhosis [55–58], characterized by elevated LVEF and lower ventricular-arterial coupling (*E*_*a*_/*E*_es_) [59]. This discrepancy may arise from differences in time scales: hyperdynamic states typically result from chronic compensatory mechanisms (e.g., systemic vasodilation), whereas our model simulated acute impedance elevation without long-term neurohormonal adaptations.

The simulation revealed a disproportionate reduction in hepatic arterial flow (94.3% decline) compared to portal vein flow (51.9% reduction), which contrasts with clinical observations of preserved hepatic arterial perfusion in cirrhosis [60]. This discrepancy suggests that our model underestimated compensatory mechanisms, particularly the hepatic arterial buffer response (HABR) [61], a critical neurohumoral autoregulatory process that mitigates portal flow reductions [62]. Future models should integrate HABR dynamics to improve the accuracy of dual blood supply simulations. Chloe et al. [27], in pig-based closed-loop models of liver resection, reported increases in portal vein pressure and pressure drop, as well as decreases in hepatic artery and portal vein flows. Our simulations reproduce the same trends. Similarly, Chloe et al. [27], in rat-based closed-loop models incorporating 3D liver vascular geometry from corrosion casting and μ-CT imaging, captured chronic effects such as increases in arterial pressure and SW. While their models were calibrated to species-specific measurements, our study uses literature-reported human parameters.

Persistent preload reduction and afterload elevation caused by hepatic impedance may induce ventricular remodeling, contributing to cirrhotic cardiomyopathy [51]. In surgical contexts such as hepatectomy or liver transplantation, cardiac function is a critical determinant of perioperative risk and outcomes [64–67]. Our findings, reduced CO, SV, and SW, highlight these parameters as potential predictors of intraoperative and postoperative cardiac performance, providing a theoretical basis for risk stratification and clinical intervention.

Wang et al. conducted a global sensitivity analysis of hepatic venous pressure gradient (HVPG) measurement using a stochastic hepatic circulation model, identifying presinusoidal portal and splanchnic resistances as the primary determinants of HVPG. Building on a similar approach, the present study extended the analysis to cardiac function indicators, including CO, MAP, and SW, to investigate how hepatic vascular factors influence cardiac performance under conditions of acute liver injury. The results showed that both CO and SW were consistently most sensitive to *R*_*pv_d*_, indicating that *R*_*pv_d*_ is the principal hepatic determinant of cardiac pumping capacity. Increases in *R*_*pv_d*_ reduce hepatic inflow, decrease venous return, and lower ventricular preload, directly suppressing CO and diminishing left ventricular SW. Although less dominant than portal resistance, alterations in *R*_*ha_d*_ can still modulate venous return indirectly through changes in hepatic perfusion. In contrast, MAP was most sensitive to *R*_*ha_d*_, suggesting that, within the model’s parameter range, arterial pressure is more strongly influenced by hepatic arterial hemodynamics than by *R*_*pv_d*_ or *R*_*hs*_. *R*_*hv*_ exhibited minimal sensitivity across all cardiac function metrics, indicating that hepatic venous resistance has a negligible effect on cardiac performance in the tested parameter range. Moreover, the close agreement between first-order and total-order sensitivity indices implies limited interactions among parameters, with each resistance primarily affecting cardiac function independently. Overall, variations in presinusoidal vascular resistance are the key drivers of cardiac impairment, whereas sinusoidal and venous resistances exert comparatively modest effects. This quantitative ranking of hepatic parameters provides a basis for targeted parameter calibration and patient-specific modeling, supporting the quantitative evaluation of cardiac function under acute liver injury.

Leveraging a closed-loop cardiovascular modeling framework, this study provides a approach to analyze the hemodynamic impact of acute liver injury on cardiac function, overcoming the constraints of traditional open-loop models. Current liver modeling has achieved highly refined and detailed representations [68,69], with some studies employing open-loop LPM approaches that prescribe fixed pressures at the hepatic inlet and outlet, while failing to incorporate the complete circulatory system or account for cardiac effects [26,70–72]. The hemodynamic modeling and analysis based on animal liver closed-loop LPM can provide a reference for understanding hemodynamic changes related to human liver surgery [27,63,73]. This study has several limitations: (1) The 0D lumped parameter model captured global hemodynamics but could not resolve regional microcirculatory heterogeneity; (2) Model lacked comprehensive integration of neurohormonal regulation and HABR mechanisms; (3) Simulation results require further validation within vivo or clinical datasets. Future work should focus on validating the model using patient-specific data. In patients with liver disease, non-invasive hemodynamic measurements such as left ventricular stroke volume from echocardiography [74], heart rate, and 4D-flow portal vein flow [75] are routinely available. Invasive measurements, including portal vein pressure, can also be obtained when clinically indicated. These data could be used to calibrate and personalize the model, enabling more accurate predictions of cardiac function for interventions such as TIPS procedures, liver resection, or transplantation. Furthermore, incorporate advanced imaging techniques, real-time hemodynamic monitoring, and adaptive compensatory mechanisms to refine the model’s predictive capability for cardiac function assessment in hepatic surgery.

## 5. Conclusions

This study applies a closed-loop hemodynamic modeling framework to quantitatively assess how acute liver injury alters systemic circulation and cardiac function. Simulation results revealed that as acute liver injury intensifies, the blood flow distribution within the hepatic dual blood supply system significant changes, which in turn reduce cardiac preload and increase afterload. Consequently, CO, SBP, SV, LVEF, and SW decrease, while DBP and *E*_*a*_ increase. These findings provide a novel theoretical perspective on the pathophysiological interplay between acute liver injury and cardiac function, offering important guidance for cardiovascular evaluation and pharmacological interventions in clinical settings such as liver cirrhosis and pre-transplant assessments.

## Supporting information

S1 Text Governing equations of lumped-parameter components. (DOCX)
