# Supplementary material for: Hemodynamic impact of acute liver injury on cardiac function: An in silico study via a closed-loop cardiovascular model
Source: PLoS Comput Biol. 2026 Feb 24;22(2):e1014006. doi: 10.1371/journal.pcbi.1014006 (PMC12948313; doi:10.1371/journal.pcbi.1014006)
Supplement: S1 Text — (DOCX) [file pcbi.1014006.s001.docx]

# **Appendix A.** Governing Equations of Lumped-Parameter Components

This appendix summarizes the governing equations of the 0D lumped-parameter elements used in the cardiovascular model.

## Resistance

Represents the resistance of blood vessels to blood flow, modeling the frictional and resistive effects within the vasculature.


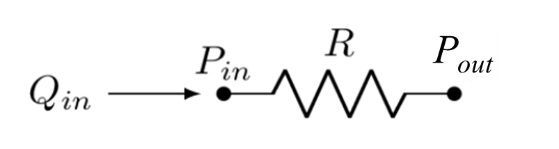

$$P_{in}-P_{out}=R\cdot Q_{in}$$

## Capacitor

Models the compliance of blood vessels or the elasticity of cardiac chambers, allowing storage of blood and smoothing of pressure fluctuations.


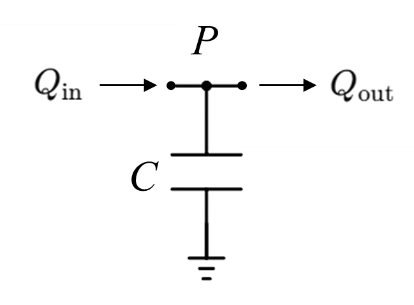

$$Q_{\text{in}}-Q_{\text{out}}=C\frac{dP}{dt}$$

## 3．Inductor

Represents the inertial effects of blood flow, capturing the dynamic response during acceleration or deceleration of blood.


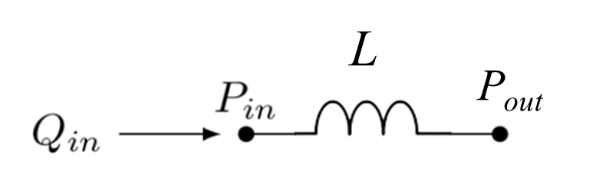

$$P_{\text{in}}-P_{\text{out}}=L\frac{dQ_{in}}{dt}$$

## Diode

Models the pressure drop across a valve with unidirectional flow, which is implemented as a non-linear hyperbolic-tangent resistor [1].


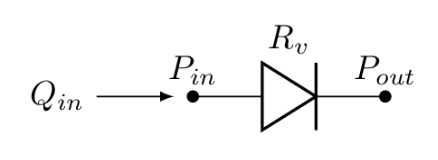

$$P_{\text{in}}-P_{\text{out}}-Q_{\text{in}}\left[R_{\min}+(R_{\max}-R_{\min})\frac{1}{2}\left(1+\tanh(k(P_{\text{out}}-P_{\text{in}}))\right)\right]=0$$

$$Q_{\text{in}}-Q_{\text{out}}=0$$

## Windkessel RCR

The Windkessel RCR is a commonly used lumped-parameter element that represents the vascular system. It combines a proximal resistance (*Rₚ*), a compliance (*C*), and a distal resistance (*R_d_*) to capture the combined effects of vascular resistance and arterial compliance on blood pressure and flow. This model effectively reproduces the pressure decay during diastole and the buffering of pulsatile blood flow by the arteries.


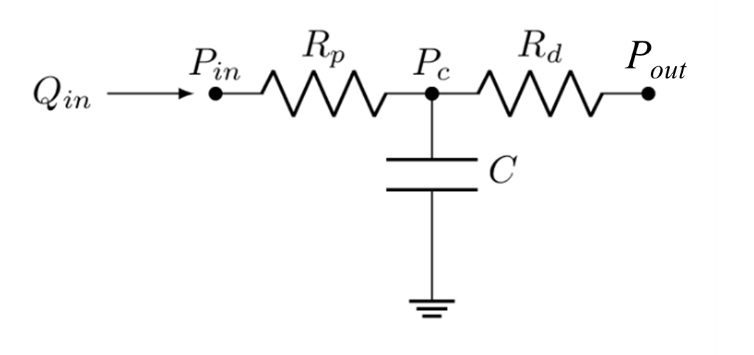

$$R_{d}Q_{\text{in}}-P_{c}+P_{out}-R_{d}C\frac{dP_{c}}{dt}=0$$

$$P_{\text{in}}-P_{c}-R_{p}Q_{\text{in}}=0$$

**References:**

1. Pfaller MR, Hörmann JM, Weigl M, Nagler A, Chabiniok R, et al. (2019) The importance of the pericardium for cardiac biomechanics: from physiology to computational modeling. Biomech Model Mechanobiol 18: 503-529.
